# Effect of Oral Supplementation of Kratom (*Mitragyna speciosa* Korth.) on Gastrointestinal Nematodes and Serum Lipid Peroxidation in Crossbred Saanen Goats

**DOI:** 10.3390/vetsci13090893

**Published:** 2026-08-31

**Authors:** Sopon Veerapan, Supawit Triwutanon, Theera Rukkwamsuk

**Affiliations:** Department of Large Animal and Wildlife Clinical Sciences, Faculty of Veterinary Medicine, Kasetsart University, Kamphaeng Saen, Nakhon Pathom 73140, Thailand; sopon.ve@ku.th (S.V.); supawit.tr@ku.ac.th (S.T.)

**Keywords:** fecal egg count, goat, kratom, malondialdehyde, *Mitragyna speciosa* Korth., oxidative stress

## Abstract

The objective of this study was to compare fecal egg count (FEC) and serum malondialdehyde (MDA) concentration in naturally parasite-infected goats following administration of an anthelmintic drug and *Mitragyna speciosa* Korth. as the control and treatment groups, respectively. A total of 34 female goats were randomly allocated into two groups. The control group received albendazole at a dose of 15 mg/kg on the first day of the experiment. Then followed by a daily placebo until day 21. The treatment group was orally administered 5 g/head of dried *Mitragyna speciosa* Korth. daily for 21 days. Hematological parameters were evaluated on days 0 and 21, while blood biochemical parameters, including blood urea nitrogen (BUN), creatinine, aspartate aminotransferase (AST), and gamma-glutamyl transferase (GGT), were measured on day 0 and at 7-day intervals until day 35. Fecal egg count and serum MDA concentration were determined on day 0 and every 7 days until day 42. The results showed that plasma protein levels were significantly affected by time (*p* < 0.05) and group (*p* < 0.05), while red blood cell count was significantly influenced by time (*p* < 0.05). No significant group effects were observed for BUN, creatinine, AST, and GGT. However, AST showed a significant effect over time (*p* < 0.05). Hematological and blood biochemical parameters remained within physiological ranges in both groups, with no evidence of kratom-associated toxicity. Log_2_-transformed fecal egg counts per gram of feces (Log_2_-EPG) showed significant time and time × group interaction effects (*p* < 0.001 and *p* = 0.008, respectively). Fecal egg count reduction test (FECRT) showed significant time and time × group interaction effects (*p* = 0.009 and *p* = 0.013, respectively). Serum MDA was significantly lower in the kratom group only at day 7 (*p* < 0.05). In conclusion, *Mitragyna speciosa* Korth. supplementation did not significantly reduce fecal egg count compared to conventional anthelmintic treatment but demonstrated a transient effect on oxidative stress, as indicated by changes in serum MDA concentration.

## Simple Summary

This study evaluated the effects of oral kratom (*Mitragyna speciosa* Korth.) supplementation on gastrointestinal nematode infection and oxidative stress in naturally infected crossbred Saanen goats. Thirty-four female goats were assigned to either an albendazole-treated control group or a kratom-supplemented group for 21 days. Fecal egg count (FEC), hematological and biochemical parameters, and serum malondialdehyde (MDA) concentrations were assessed throughout the study. Kratom supplementation did not significantly reduce FEC compared with albendazole, although a slight numerical reduction was observed. Hematological and biochemical values remained within normal physiological ranges, indicating no adverse effects from short-term supplementation. Serum MDA concentrations decreased significantly during the first week, suggesting a transient antioxidant effect attributable to kratom bioactive compounds, including mitragynine. Overall, kratom exhibited limited antiparasitic activity under the conditions of this study but may provide short-term antioxidant benefits. Further research is warranted to determine the optimal dosage, evaluate long-term safety, and assess its potential role as a complementary component of integrated parasite control strategies in small ruminants.

## 1. Introduction

Gastrointestinal nematode (GIN) infection is among the most important health challenges affecting goats and other small ruminants globally [1]. Parasitic nematodes such as *Haemonchus contortus* have been reported to negatively impact animal health and productivity by causing anemia, reduced feed intake, impaired growth, and increased susceptibility to secondary infections [2,3]. These effects result in substantial economic losses, particularly in tropical and subtropical regions where environmental conditions favor parasite survival and transmission [4].

The control of GIN infections relies largely on the use of synthetic anthelmintic drugs [5]. However, the intensive and prolonged use of these compounds has led to the widespread development of anthelmintic resistance, reducing their effectiveness and threatening the sustainability of small ruminant production systems [6]. Consequently, there is increasing interest in alternative or complementary parasite control strategies, including the use of medicinal plants with potential anthelmintic and antioxidant properties [5].

Parasitic infections are also known to induce oxidative stress in the host, partly through increased production of reactive oxygen species associated with inflammation and tissue damage [7]. Lipid peroxidation, commonly assessed by markers such as malondialdehyde (MDA), is an important indicator of oxidative damage to cell membranes and has been linked to reduced health and performance in parasitized animals [8]. Changes in MDA concentrations should be interpreted as indicators of oxidative status rather than as direct evidence of reduced parasite-associated oxidative damage, due to the fact that MDA is a nonspecific marker that can be influenced by various physiological and pathological processes. Plant-derived antioxidants may help mitigate oxidative stress while simultaneously contributing to parasite control, offering a dual benefit for animal health.

Kratom (*Mitragyna speciosa* Korth.), a plant belonging to the Rubiaceae family and native to Southeast Asia, has been traditionally used for various medicinal purposes [9]. The leaves of kratom contain a diverse array of bioactive compounds, particularly indole alkaloids, flavonoids, and phenolic compounds [10]. These constituents have been reported to possess antimicrobial, anti-inflammatory, antiparasitic, and antioxidant activities [11]. Despite growing scientific interest in kratom, limited information is available regarding its effects on gastrointestinal nematodes in small ruminants, especially under in vivo conditions.

It was hypothesized that kratom supplementation would reduce GIN infection and blood lipid peroxidation in goats. Therefore, the objective of this study was to evaluate the effects of kratom on fecal egg counts and serum malondialdehyde (MDA) concentrations in goats. This research aimed to provide insight into the potential role of kratom as a natural anthelmintic and antioxidant agent, contributing to the development of sustainable and integrated parasite management strategies in small ruminant production systems.

## 2. Materials and Methods

### 2.1. Experimental Animals and Study Design

The experiment was conducted in the demonstration farm, located at Kamphaeng Saen, Nakhon Pathom, Thailand (14.0228° N, 99.9783° E). The goats were maintained in an elevated housing facility raised above ground level. The floor consisted of concrete slatted panels that allowed feces and urine to pass through the gaps, thereby improving sanitation and reducing moisture accumulation. The building was divided into several smaller pens to manage the animals according to their experimental groups. The housing system was designed as an open-sided structure, providing natural ventilation and environmental exposure under typical farm management conditions. The goats housed in the experiment were thirty-four goats naturally infected with gastrointestinal nematodes. Animals were managed under the same housing and feeding conditions throughout the study period. The diet comprised cassava chips, soybean hull pellets, palm kernel meal, sweet potato, soybean meal, commercial pellets containing 21% crude protein, and molasses. The goats were randomly allocated into two experimental groups (n = 17 per group): a control and a treatment group. The average body weights of the goats at the start of the experiment were 40.90 ± 5.82 kg for the control and 40.44 ± 3.29 kg for the treatment group, respectively. The control goats were orally drenched with albendazole (Abentel, Atlantic Laboratories Corporation Ltd., Samut Prakan, Thailand) at a dosage of 15 mg/kg of body weight on day 1 of the experimental period.

The experimental period lasted 21 days, with the respective treatments administered daily. Treatment administration ceased on Day 21, whereas the post-treatment observation period continued until Day 42. Fecal and blood samples were collected at predefined 7-day intervals to evaluate gastrointestinal nematode infection, biochemical parameters, and oxidative stress status during and after the treatment period.

### 2.2. Dried Kratom

Fresh kratom leaves were obtained from a certified farm in Surat Thani province. Dry matter (DM) was determined using the microwave test method [12]. Fresh kratom leaves had a DM content of 30%, whereas the dried leaves contained 56.06% condensed tannins and 2.05% mitragynine on a DM basis. Fresh kratom leaves were dried using a hot-air oven at 40 °C for 2 days until the moisture content was reduced to below 5%. Dried kratom leaves were ground into a fine powder and stored at room temperature in a dark room until use. Condensed tannin was determined using the method described previously [13]. The mitragynine content used in this study was confirmed according to the official certificate provided by the farm source.

### 2.3. Treatment Administration

During 21 days of treatment period, goats in the treatment group received 5 g/head of kratom powder mixed with 30 mL of placebo, whereas goats in the control group received albendazole on day 1, followed by a placebo daily until day 21. The placebo consisted of 2% guar gum. Treatments were administered according to the experimental protocol, and all animals were monitored daily for general health and adverse reactions throughout the study period. The treatment group received 2.55 ± 0.20 mg/kg of BW of mitragynine and 0.07 ± 0.01 g/kg of BW of condensed tanning, respectively. The dosages of mitragynine ranged from 2.15 to 2.99 mg/kg of BW, while the dosages of condensed tannin ranged from 0.05 to 0.08 g/kg of BW.

### 2.4. Fecal Sample Collection and Egg Count Analysis

Fecal samples were collected directly from the rectum of each goat on days 0, 7, 14, 21, 28, 35, and 42 of the experiment. Samples were stored in airtight containers and analyzed within 24 h of collection.

Fecal egg counts (FEC) were determined and expressed as eggs per gram (EPG) of feces using a standard quantitative flotation technique. Changes in EPG over time were used to assess the effect of kratom on gastrointestinal nematode infection. The fecal egg count reduction test (FECRT) was also used for analysis. The formulate of EPG and FECRT is below;

$$EPG=\left(Chamber\;1+Chamber\;2\right)\times 50$$

$$FECRT\%=\left(\frac{Post-treatment}{Pre-treatment}-1\right)\times 100$$

### 2.5. Blood Sample Collection

Blood samples were collected via jugular venipuncture on the designated sampling days. Samples were divided into tubes containing anticoagulant for hematological analysis and plain tubes for serum or plasma separation. Serum and plasma were obtained by centrifugation and stored at appropriate temperatures until analysis.

### 2.6. Hematological and Blood Chemistry Analysis

Hematological parameters were determined using standard laboratory procedures. Blood biochemical analyses were performed to assess kidney and liver function, including blood urea nitrogen (BUN), creatinine, plasma protein concentration, aspartate aminotransferase (AST), and gamma-glutamyl transferase (GGT). All analyses were conducted using established diagnostic methods and commercial assay kits according to the manufacturers’ instructions.

### 2.7. Serum Lipid Peroxidation Assay

Lipid peroxidation in serum samples was assessed using the thiobarbituric acid reactive substances (TBARS) method. This assay measures MDA levels as an indicator of oxidative damage to lipids. The content of TBARS in serum was determined by modification of the procedure described by [14]. Thawed goat serum (510 µL) was mixed with 170 µL of 10% trichloroacetic acid (TCA) and then centrifuged at 5000× *g* for 5 min. An aliquot of 300 µL of the supernatant was mixed with 300 µL of 0.8% thiobarbituric acid (TBA). The mixture was incubated at 97 °C for 1 h, and the reaction was stopped by placing the samples on ice for 30 min. Centrifuge at 1500× *g* for 10 min at 4 °C. The absorbance of the supernatant was measured using a spectrophotometer at a wavelength of 532 nm. Compared absorbance with a 1,1,3,3-tetramethoxypropane standard curve.

### 2.8. Statistical Analysis

Data from one goat from the treatment group was excluded from the analysis because the goat was diagnosed with cervical vertebrae dislocation. The fecal egg count per gram was transformed by Log_2_ as Log_2_-transformed fecal egg count per gram (Log_2_-EPG). The normality of data distribution was assessed using the Shapiro-Wilk test and the Kolmogorov-Smirnov test. Data were expressed as mean ± standard error. Differences between control and treatment groups and changes over time were analyzed. Hematological parameters, BUN, AST, GGT, Log_2_-EPG, and FECRT were analyzed using repeated measures ANOVA; creatinine and malondialdehyde concentrations were analyzed using repeated measures ANCOVA. When a significant group effect was identified, pairwise comparisons between groups at each time point were conducted using the Bonferroni post hoc test to determine specific differences. Statistical significance was set at *p* < 0.05.

## 3. Results

### 3.1. Hematology

The hematological and biochemical profiles of goats in the control and treatment groups at Day 0 and Day 21 are presented in Table 1. No significant group effect was observed for red blood cell (RBC) counts (*p* = 0.502). However, a significant effect of time was detected, as indicated by differing superscripts, suggesting changes in RBC counts within groups over the study period. Similarly, pack cell volume (PCV) did not differ significantly between groups (*p* = 0.292). The absence of differing superscripts within each group indicated that PCV remained stable from Day 0 to Day 21, with no significant within-group variation. In contrast, plasma protein concentration was significantly affected by treatment (*p* = 0.031), with higher values observed in the treatment group compared with the control group. A significant time effect was also detected, indicating that plasma protein levels changed from baseline to Day 21 in both groups. For white blood cell (WBC) counts, no significant effects of group or time were observed (*p* = 0.174), and values remained comparable between Day 0 and Day 21 within each group.

### 3.2. Blood Biochemistry

The effects of treatment on serum biochemical parameters across the experimental period are presented in Table 2. Blood urea nitrogen (BUN) concentrations were not significantly affected by time (*p* = 0.319), and no significant differences between the control and treatment groups were observed at any sampling point, as indicated by identical superscripts. Serum creatinine levels were also not significantly influenced by time (*p* = 0.152). However, a group effect was observed at several sampling points, with the treatment group generally exhibiting lower creatinine concentrations than the control group, as indicated by differing superscripts. Despite these differences, no consistent temporal trend in creatinine levels was evident within either group. Overall, BUN and creatinine levels remained relatively stable throughout the study in both groups. Aspartate aminotransferase (AST) activity showed a significant effect of time (*p* < 0.001), with fluctuations observed across sampling days in both groups. However, no significant differences between the control and treatment groups were detected at corresponding time points. For gamma-glutamyl transferase (GGT), no significant effect of time was observed (*p* = 0.327). Enzyme activity remained stable from Day 0 to Day 35, with no significant differences between groups.

### 3.3. Fecal Egg Count and Fecal Egg Count Reduction Test

Log_2_-transformed fecal egg counts per gram of feces (Log_2_-EPG) in the control and treatment groups throughout the experimental period are presented in Table 3. At baseline (Day 0), mean log_2_-EPG values were comparable between groups. From Day 7 to Day 42, log_2_-EPG values showed minor fluctuations in both groups but remained within a similar range. FECRT in the control and treatment groups are presented in Table 4.

### 3.4. Malondialdehyde

Serum MDA concentrations in the control and treatment groups during the experimental period are presented in Table 5. At Day 0, the treatment group exhibited significantly higher MDA levels than the control group. A repeated-measures ANCOVA was applied to adjust for baseline (Day 0). By Day 7, serum MDA concentrations in the treatment group had decreased markedly and were significantly lower than those in the control group. From Day 14 to Day 42, no significant differences in serum MDA concentrations were observed between the two groups. In both groups, serum MDA levels fluctuated throughout the study period, with a significant effect of sampling day detected (*p* < 0.001). Overall, MDA concentrations showed a declining trend toward the end of the experimental period in both groups.

## 4. Discussion

Hematological indices and blood biochemical markers remained largely stable throughout the study. Parameters associated with liver and kidney function, including aspartate aminotransferase (AST), gamma-glutamyl transferase (GGT), blood urea nitrogen (BUN), and creatinine, remained within their normal physiological ranges, and no adverse trends related to supplementation were observed. From a practical perspective, the absence of detrimental health effects is particularly important, as safety is a fundamental prerequisite for the implementation of plant-based interventions in livestock production systems. Previous studies have demonstrated that methanolic extracts of kratom leaves can induce acute hepatotoxicity and nephrotoxicity in rodents after 14 days of administration at doses of 100, 500, and 1000 mg/kg body weight [15]. In contrast, supplementation with dried kratom leaf powder for 14 days did not adversely affect the hematological profile of goats. Moreover, goats receiving dried kratom leaf powder exhibited significantly lower concentrations of NH_3_-N and BUN (*p* < 0.05 and *p* < 0.01, respectively) [16]. These findings suggest that the physiological effects of kratom may differ considerably depending on the animal species, dosage, preparation method, and duration of administration. However, a reduction in red blood cell (RBC) count was observed in the treatment group on day 21. This finding may be explained by the different mechanisms of action of conventional anthelmintic drugs and plant-derived phytochemicals. In the control group, albendazole effectively eliminated adult gastrointestinal nematodes, thereby reducing blood loss caused by hematophagous parasites such as *Haemonchus contortus*, which is one of the most prevalent GIN species infecting goats. In contrast, kratom supplementation may not directly eliminate adult parasites, allowing continued blood feeding and subsequent erythrocyte loss. Furthermore, parasite-derived hemolytic factors may contribute to erythrocyte destruction through disruption of erythrocyte membranes [17]. Variations in AST concentrations may also reflect a range of physiological processes, including hemolysis, muscle metabolism, and hepatic activity, rather than liver injury alone [18].

The present study demonstrated that supplementation with *Mitragyna speciosa* Korth. leaves did not result in a statistically significant difference in fecal egg count (FEC) between the treatment and control groups. Nevertheless, although the difference was not statistically significant, a numerical reduction in eggs per gram (EPG) was observed in the kratom-supplemented group, suggesting a possible but limited biological effect. In the control group, the log_2_-EPG value changed only slightly, from 10.80 ± 1.64 on day 0 to 10.82 ± 1.38 on day 14, indicating that the gastrointestinal nematode population in this herd was likely resistant to albendazole treatment. To further support this observation, the fecal egg count reduction test (FECRT) was performed. Previous in vivo studies in sheep, goats, and deer have shown that the amount of condensed tannins required to exert antiparasitic effects is approximately 0.9–1.2 g/kg BW of goat, corresponding to about 3–4% of dietary dry matter [19]. Likewise, other studies have demonstrated that the antiparasitic activity of tannin-containing plants is dose-dependent, particularly in experiments evaluating the efficacy of quebracho extracts against gastrointestinal parasites in sheep [20]. In contrast, de Fátima França et al. [21] reported that supplementation with condensed tannin extracts at concentrations ranging from 1% to 6% of dietary dry matter did not result in significant differences among treatment groups, indicating that the response may vary according to experimental conditions, parasite species, tannin source, and dosage. Considering that goats generally consume dry matter equivalent to approximately 3–4% of their body weight, the effective dose proposed by Hoste et al. [19] would correspond to approximately 0.9 g of tannins per kilogram of body weight. In the present study, the treatment group received condensed tannin at a dosage of 0.07 ± 0.01 g/kg of BW, which was substantially lower than the effective dose reported previously [19]. Therefore, the amount of tannins provided by kratom leaf supplementation in the present study may have been insufficient to achieve a consistent antiparasitic effect.

Despite the absence of statistically significant differences, FECRT showed that EPG values decreased by more than −30% per week after seven days of supplementation, with the greatest reduction (−46%) observed on day 21. Furthermore, fecal egg excretion remained lower for one week after supplementation had ceased, suggesting a possible residual or carry-over effect. However, the response gradually diminished over time, indicating that continuous supplementation may be necessary to maintain its antiparasitic activity. Because the concentration of condensed tannins in the kratom supplement was relatively low, it is possible that the observed reduction in EPG and the FECRT results were attributable, at least in part, to the biological activity of mitragynine and other phytochemicals present in the leaves. Nevertheless, further studies are required to clarify the relative contributions of these compounds and their underlying mechanisms of action. From a practical perspective, the findings of the present study indicate that kratom leaf supplementation at the inclusion rate tested is unlikely to provide sufficient antiparasitic activity to replace conventional anthelmintic treatment.

Malondialdehyde (MDA), a well-established biomarker of lipid peroxidation measured using the thiobarbituric acid reactive substances (TBARS) assay, differed significantly between the treatment and control groups during the first week of supplementation. However, no significant differences were detected thereafter. This temporal pattern suggests an acute physiological response to the phytochemical constituents of *Mitragyna speciosa* leaves, which may initially influence oxidative pathways but fail to maintain a sustained effect over time. This interpretation is supported by previous studies demonstrating the antioxidant properties of *Mitragyna speciosa* extracts. In diabetic rat models receiving different concentrations of kratom leaf extract, a significant reduction in MDA concentrations was observed, suggesting attenuation of oxidative stress associated with metabolic disorders [22]. Similarly, kratom extracts have shown strong antioxidant activity in DPPH, ABTS, and ferric reducing antioxidant power (FRAP) assays, indicating their ability to scavenge reactive oxygen species (ROS) and limit oxidative damage [23].

Nevertheless, accumulating evidence indicates that repeated exposure to the bioactive compounds present in kratom may induce physiological adaptation. For example, repeated administration of 7-hydroxymitragynine, one of the principal alkaloids found in kratom leaves, has been reported to produce tolerance to its antinociceptive effects [24]. Likewise, in aquatic models, supplementation with a high dose of kratom extract (50 mg/kg body weight) in *Nile tilapia* reduced oxidative stress through modulation of acetylcholinesterase activity, thereby influencing neuromuscular transmission and inflammatory responses [25]. However, differences in dosage, route of administration, and species-specific physiology limit the direct extrapolation of these findings to ruminants. Furthermore, surveys conducted among kratom users in the United States have reported the occurrence of tolerance, withdrawal symptoms, and perceived dependence, although these effects were generally considered mild [26]. Consistent with these findings, data from the Princess Mother National Institute on Drug Abuse Treatment indicate that regular kratom consumption may lead to the gradual development of tolerance, resulting in progressively higher intake levels over time. However, the mechanisms of mitragynine remained speculative due to the absence of direct pharmacokinetic measurements in this study. Taken together, the findings of the present study suggest that kratom leaves may exert antioxidant effects during the early phase of supplementation. However, the diminishing effect observed over time indicates that physiological adaptation to the bioactive compounds may occur, thereby reducing their long-term efficacy.

## 5. Conclusions

Dietary supplementation with *Mitragyna speciosa* Korth. leaves at the tested inclusion rate did not significantly reduce fecal egg counts, indicating limited antiparasitic efficacy, although a minor numerical reduction in EPG suggests a possible biological effect. Kratom supplementation also transiently reduced MDA concentrations during the first week, suggesting short-term antioxidant activity that was not sustained over time. Hematological and serum biochemical parameters remained within physiological ranges, with no adverse effects observed, indicating that dried kratom leaves were safe for short-term dietary inclusion at the tested level. Overall, kratom leaves appear to have limited antiparasitic efficacy but potential transient antioxidant effects. Further studies should evaluate higher inclusion rates, optimized tannin concentrations, and longer supplementation periods while monitoring safety and animal health.

## Figures and Tables

**Table 1:** Hematological parameters of animals in the control (n = 17) and treatment (n = 16) groups at Day 0 and Day 21 following administration. Values are presented as mean ± standard deviation (SD). Different superscript letters within the same column indicate significant differences between sampling times within the same group (*p* < 0.05). Differences between the control and treatment groups at the same sampling time were analyzed separately, and corresponding *p*-values are shown.

Parameter	Experimental Group		*p*-Value		
	Control	Treatment	Time Effect	Group Effect	Interaction
Red blood cells (10^6^/µL)					
Day 0	13.93 ± 1.78 ^a^	13.52 ± 1.83 ^a^	0.005	0.502	0.927
Day 21	13.35 ± 1.95 ^b^	12.89 ± 1.97 ^b^			
Pack cell volume (%)					
Day 0	23.13 ± 2.48 ^a^	21.94 ± 2.14 ^a^	0.565	0.292	0.381
Day 21	22.53 ± 2.23 ^a^	22.06 ± 2.84 ^a^			
Plasma protein (g/dL)					
Day 0	7.17 ± 0.68 ^a^	7.44 ± 0.54 ^a^	0.001	0.031	0.130
Day 21	7.37 ± 0.49 ^b^	7.91 ± 0.52 ^b^			
White blood cells (10^3^/µL)					
Day 0	10.80 ± 3.25 ^a^	10.18 ± 2.69 ^a^	0.185	0.174	0.206
Day 21	12.32 ± 3.64 ^a^	12.22 ± 2.49 ^a^			

**Table 2:** Blood biochemical parameters of animals in the control (n = 17) and treatment (n = 16) groups measured at Day 0, 7, 14, 21, 28, and 35 during the experimental period. Values are expressed as mean ± standard deviation (SD). The *p*-values represent the effect of sampling day within each parameter across the experimental period.

Parameter	Experimental Group	Sampling Day						*p*-Value		
		Day 0	Day 7	Day 14	Day 21	Day 28	Day 35	Time Effect	Group Effect	Interaction
BUN (mg/dL)	Control	15.43 ± 2.36	15.00 ± 3.45	15.12 ± 2.77	15.24 ± 3.49	14.00 ± 0.57	15.06 ± 2.37	0.319	0.325	0.787
	Treatment	16.34 ± 2.56	15.16 ± 2.41	15.94 ± 3.00	14.68 ± 2.72	14.86 ± 2.08	15.61 ± 2.37			
Creatinine (mg/dL)	Control	1.16 ± 0.28	1.09 ± 0.34	1.28 ± 0.24	1.21 ± 0.33	1.16 ± 0.37	1.14 ± 0.24	0.789	0.464	0.438
	Treatment	0.93 ± 0.16	1.01 ± 0.28	1.06 ± 0.19	0.99 ± 0.26	0.92 ± 0.27	1.14 ± 0.30			
AST (U/L)	Control	43.64 ± 7.48	54.74 ± 13.93	42.72 ± 8.16	42.81 ± 5.39	42.59 ± 12.40	50.46 ± 5.39	<0.001	0.953	0.260
	Treatment	41.85 ± 8.49	52.09 ± 8.66	48.86 ± 11.25	42.01 ± 9.45	42.63 ± 6.84	48.59 ± 6.86			
GGT (U/L)	Control	31.64 ± 11.06	27.96 ± 8.93	35.02 ± 6.13	34.13 ± 3.12	33.93 ± 4.35	32.25 ± 6.55	0.327	0.446	0.260
	Treatment	38.24 ± 4.88	34.19 ± 8.17	32.30 ± 9.21	32.98 ± 7.46	32.39 ± 6.3	34.27 ± 9.00			

**Table 3:** Log_2_-transformed fecal egg counts per gram of feces (log_2_-EPG) in animals from the control (n = 17) and treatment (n = 16) groups measured at Day 0, 7, 14, 21, 28, 35, and 42 during the experimental period. Values are presented as mean ± standard deviation (SD). The *p*-value represents the overall effect of sampling day across the study period.

Experimental Group	Log_2_-EPG							*p*-Value		
	Day 0	Day 7	Day 14	Day 21	Day 28	Day 35	Day 42	Time Effect	Group Effect	Interaction
Control	10.80 ± 1.64	9.87 ± 1.53	10.82 ± 1.38	10.79 ± 1.12	10.69 ± 1.35	10.93 ± 1.19	10.62 ± 1.28	<0.001	0.974	0.008
Treatment	11.23 ± 1.14	10.59 ± 1.13	10.75 ± 1.35	10.36 ± 1.14	10.44 ± 1.27	10.80 ± 1.41	10.45 ± 1.39			

**Table 4:** Fecal egg count reduction test (%) of animals from the control (n = 17) and treatment (n = 16) groups measured at Day 0, 7, 14, 21, 28, 35, and 42 during the experimental period. Values are presented as mean ± standard deviation (SD). The *p*-value represents the overall effect of sampling day across the study period.

Experimental Group	Fecal Egg Count Reduction Test (%)						*p*-Value		
	Day 7	Day 14	Day 21	Day 28	Day 35	Day 42	Time Effect	Group Effect	Interaction
Control	−56.1 ± 27.4	−17.2 ± 54.6	−15.3 ± 46.1	−15.7 ± 35.5	−12.7 ± 38.5	−25.3 ± 38.5	0.009	0.250	0.013
Treatment	−36.5 ± 22.5	−33.0 ± 22.7	−46.3 ± 18.8	−40.7 ± 31.4	−25.5 ± 30.8	−35.2 ± 44.1			

**Table 5:** Serum MDA concentrations in the control (n = 17) and treatment (n = 16) groups measured at Day 0, 7, 14, 21, 28, 35, and 42 during the experimental period. Values are expressed as mean ± standard deviation (SD). Different superscript letters within the same column indicate significant differences between the control and treatment groups at the same sampling time (*p* < 0.05). The *p*-value represents the overall effect of sampling day during the study period.

Experimental Group	Serum MDA Concentration (µM)							*p*-Value		
	Day 0	Day 7	Day 14	Day 21	Day 28	Day 35	Day 42	Time Effect	Group Effect	Interaction
Control	4.565 ± 1.445	4.896 ± 1.107 ^a^	4.782 ± 2.345 ^a^	5.668 ± 3.206 ^a^	6.415 ± 2.004 ^a^	5.699 ± 2.908 ^a^	3.609 ± 0.822 ^a^	0.524	0.037	0.528
Treatment	6.816 ± 1.786	2.299 ± 0.595 ^b^	5.427 ± 1.926 ^a^	5.027 ± 2.163 ^a^	6.104 ± 2.239 ^a^	4.965 ± 1.944 ^a^	3.002 ± 0.989 ^a^			

## Data Availability

The original contributions presented in this study are included in the article. Further inquiries can be directed to the corresponding author.
